# Defecation Dysfunction and Exercise Habits among Survivors of Rectal Cancer: A Pilot Qualitative Study

**DOI:** 10.3390/healthcare10102029

**Published:** 2022-10-15

**Authors:** Hiromi Nakagawa, Hiroyuki Sasai, Kiyoji Tanaka

**Affiliations:** 1 School of Nursing, Takarazuka University, Osaka 530-0012, Japan; 2 Research Team for Promoting Independence and Mental Health, Tokyo Metropolitan Institute of Gerontology, Tokyo 173-0015, Japan; 3 Faculty of Health and Sport Sciences, University of Tsukuba, Tsukuba 305-8577, Japan

**Keywords:** rectal cancer, survivors, exercise, low anterior resection syndrome, quality of life

## Abstract

This pilot qualitative study aimed to investigate exercise habits and assess defecatory dysfunction among adult survivors of rectal cancer with and without stomas. Patients were eligible for the study if they had stage I–IV rectal cancer, and less than 5 years had elapsed since surgery. We conducted semi-structured interviews with outpatients visiting two general hospitals in Japan and inquired about their diets, defecation, and exercise habits. The interview data were transcribed verbatim, interpreted, and abstracted to generate coding units; we divided the responses into categories and subcategories. Eleven patients had stomas inserted after surgery while six did not. Content analysis identified four categories common to patients with and without stomas: [diet control], [coping with defecation dysfunction], [compromising with defecation dysfunctions], and [maintenance of exercise habits]. Our results suggest the need for intervening among rectal cancer survivors to address eating habits to alleviate defecation dysfunction and exercise habits to maintain physical function. In clinical practice, symptom relief and exercise instruction may improve the well-being of cancer survivors with bowel dysfunction.

## 1. Introduction

Colorectal cancer is the third most common malignancy in the world, with more than 1.9 million new cases diagnosed in 2020. Data from 2018 showed that the prevalence of rectal cancer in Japan (*n* = 51,005) was increasing consistent with a steady rise in estimated number of patients over the past 40 years [1]. The 5-year relative survival rate of patients with rectal cancer in Japan (71.8%) is higher than that of patients with other types of cancers [2]. Moreover, the number of colorectal cancer survivors is increasing given the country’s super-aging society. Therefore, the maintenance of activities of daily living and improvement in the quality of life (QoL) for survivors of this disease is increasingly important. Strategies targeted toward managing aging, the primary cause of reduced QoL, and addressing defecation dysfunction after rectal cancer surgery are urgent unmet needs [3]. 

The intake of soluble dietary fiber can improve bowel movement [4], while exercise can increase the contractile force of pelvic floor muscles [5]. These two steps have been reported to effectively alleviate defecation dysfunction such as fecal incontinence and constipation; however, lifestyle studies of rectal cancer survivors that address diet, bowel movements, and exercise habits are scarce. Therefore, this pilot qualitative study aimed to investigate the prevalence of defecation dysfunction and the adoption of exercise habits among survivors of rectal cancer, including those with and without stomas. The importance of our study lies in its provision of fundamental clinical evidence supporting the promotion of nutritional education and physical activity in cancer survivors who experience postoperative defecation dysfunction.

## 2. Materials and Methods

### 2.1. Study Design, Setting, and Participants

This pilot study adopted a qualitative, descriptive design using semi-structured interviews. The participants were outpatients who were visiting two general hospitals in Japan. Patients were eligible for inclusion in the study if they had stage I–IV rectal cancer per the TNM classification, and less than 5 years had elapsed since surgery. The exclusion criteria were current or previous radiotherapy, psychiatric disorders, severe arrhythmias, undergoing dialysis, or cognitive dysfunction at the time of registration. The research collaborator, a colorectal surgeon, randomly selected participants from among his outpatients. The ethics committees of Takarazuka University and the participating research centers approved the study, and written informed consent was obtained from the patients via post before collecting data from medical records and interviews. The survey period was from 8 March 2021 to 30 April 2022.

In this study, ‘lifestyle’ refers to diet, bowel movement, and exercise habits. Defecation dysfunction refers to constipation, diarrhea, fecal incontinence, or defecation urgency symptoms caused by colorectal and anal problems.

### 2.2. Data Collection Procedures

#### 2.2.1. Interviews

The recruited survivors took part in pre- and postoperative semi-structured interviews for approximately 40 minutes. The investigator conducted the interviews in a single outpatient room with strict infection control for COVID-19 prevention and adequate privacy. The interviews were recorded with a Dictaphone and covered dietary, bowel movement, and exercise habits. The interview guide mainly contains items as follows: pre- and postoperative changes in eating, bowel movement, and exercise habits as well as the awareness and attitudes of the participant toward any such changes; difficulties with eating, defecation, and exercise; and support required from family and social resources (i.e., hospitals and welfare services).

The interview data were transcribed, repeatedly reviewed, and summarized. Subsequently, content analysis was conducted to identify defecation difficulties and exercise habits for each patient.

#### 2.2.2. Medical Records

Outpatient nurses, who served as research collaborators, collected the following information from the medical records: age, sex, height and weight, presence or absence of a stoma, tumor stage, date of surgery, surgical procedures, use of chemotherapy or radiotherapy, hemoglobin A1c (HbA1c) levels, and medical and smoking histories. 

#### 2.2.3. QoL Survey

The Japanese version of the ‘European Organisation for Research and Treatment of Cancer’ QoL questionnaire-30 (version 3; EORTC QLQ-30) [6,7] was used after obtaining approval from the EORTC, the organization that owns the copyright of the QoL questionnaire. The EORTC QLQ-30 is the most internationally recognized questionnaire used to investigate QoL in patients with cancer. Higher “global QoL” and functional EORTC QLQ scale values indicate more favorable patient conditions, while higher symptom scale values indicate worse conditions. The QoL survey can be used to scientifically evaluate physical and functional disability after rectal cancer surgery, both objectively and subjectively.

The participants responded to the QLQ items at the start of the interview. Information on the QoL of participants was derived from the functional and symptom scales incorporated into the QLQ and ‘global health status’ assessments.

### 2.3. Statistical Analysis

We summarized the demographic data obtained from medical records and information from the EORTC QLQ-30 data using Microsoft Excel 2019 (Microsoft Corp., Redmond, WA, USA).

The interviews were transcribed verbatim and then interpreted while preserving the context. Next, the interview data were abstracted by initial coding of the raw data to generate coding units that accurately reflected the content. We then devised subcategories by combining the identified coding units, and performed inductive categorization by combining information for each context and labeling them. Categories, subcategories, and codes were indicated by [], <>, and ““, respectively. The investigator, under supervision, consulted with an expert on qualitative research analysis to improve the accuracy of data collection and analysis.

## 3. Results

### 3.1. Participant Characteristics

The pre- and postoperative lifestyle characteristics (*n* = 17) are shown in Table 1 and Table 2. The frequency of postoperative exercise was higher than that of preoperative exercise. Five of the six patients without stomas exercised before and after surgery. Three patients with stomas did not exercise preoperatively but did do so postoperatively. The exercise duration increased after surgery in both groups. Only one participant with a stoma who exercised preoperatively no longer did so postoperatively.

### 3.2. QoL Survey

The EORTC QLQ-30 scores are shown in Table 3. The QoL of the non-stoma group was higher than that of the stoma group, with summary scores of 90 vs. 71 and global health status scores of 83 vs. 40.

### 3.3. Survey of Eating Habits

We identified five subcategories of eating habits in the non-stoma group; these were then combined into two broad categories: [diet control] and [changes in appetite] (Table 4). Under [diet control], one participant stated “*My HbA1c was approximately 6.5. HbA1c levels improved after carbohydrate and calorie restriction*”; this fell under the subcategory of <carbohydrate and calorie restriction to normalize blood glucose levels>. Another participant stated “*Water intake and various diets reduced constipation. I have constipation after dehydration due to exercise. Fishing has been my passion, and I fast for approximately 12 h the day before fishing. After trial and error, I stopped eating just before fishing so that I do not have to go to the washroom frequently. I continue to do this*”; this participant fell under the <water and dietary intake considering excretion> subcategory. Another participant who stated “*Because I had problems with defecation, I did some research on the effects of changes in diet using my PC*” belonged under <utilization of information on postoperative diet>.

With respect to the [changes in appetite] category, one participant stated “*I had no appetite due to nausea during anticancer treatment*”; this reflected the <anorexia as a side effect of chemotherapy> subcategory. In contrast, another patient who stated “*I gained weight because I did not exercise, and my dietary intake remained unchanged*” reflected<recovery of body weight>.

In the stoma group, five subcategories of eating habits were identified and combined into two broad categories: [diet control] and [taste disorders as a side effect of chemotherapy] (Table 5). One participant who said “*I try to have soluble vegetable fiber and avoid root vegetables*” reflected the <avoidance of foods high in insoluble fiber> subcategory. An example of <dietary control after colostomy> was the response of a participant whose ileus improved: “*I follow the directions and try to keep hydrated after taking laxatives,”* as this reflected the participant’s consideration of bowel movements. As an example of [taste disorders as a side effect of chemotherapy], one participant responded *“Although I had severe taste abnormality, I can still slightly taste food. I used to be fatty. My body weight was about 80 kg. I lost about 15 kg,”*; in this patient, it was considered that <taste disorders caused by chemotherapy> led to <weight loss due to reduced dietary intake>.

### 3.4. Survey of Bowel Habits

In the non-stoma group, six subcategories of bowel habits were identified. These were combined into three broad categories: [coping with defecation dysfunctions], [difficulty coping with defecation dysfunctions], and [compromising with defecation dysfunctions] (Table 4). Regarding [coping with defecation dysfunctions], one participant in the non-stoma group responded “*My understanding of defecation rhythm has improved in the 2 years after surgery*”; this reflected <understanding of defecation rhythm using a stool diary>. Regarding fecal incontinence, one participant stated “*I had a horrible experience in the field. I managed to hold it until I found a washroom. I had a passage of gas from the stoma and slight fecal incontinence. I realized that I needed to carry extra disposable diapers. I still carry a set of extra disposable diapers. That makes me feel secure*”; this reflected <carrying disposable diapers for fecal incontinence>.

In the [difficulty coping with defecation dysfunctions] category, one participant stated “*I have problems with bowel function. For example, I want to control bowel movements to at most once an hour. Currently, I have problems with bowel control such as frequent events with excessive feces. Therefore, I want to improve bowel control,*” reflecting <dissatisfaction with bowel control>; the participant also stated “*I did some research by searching the Internet,*” reflecting <a lack of information on defecation dysfunctions>.

Regarding [compromising with defecation dysfunctions], one participant responded “*What I regret most is that I did not have my large intestine checked at regular health checkups because it was embarrassing. If I had had it checked, I would not have gotten cancer. I was lucky that my cancer was accidentally found*,” reflecting <regret for their late visit to the hospital and gratitude for the treatment>. Another participant responded “*Maybe I made myself get used to it. Sometimes I feel very sad, and sometimes I accept it. That makes me feel better*,” reflecting <adaptation to defecation dysfunctions> and <coping with defecation dysfunctions>.

In the stoma group, 10 subcategories of bowel habits were identified. These were combined into four broad categories: [coping with defecation dysfunctions], [perceived difficulty in stoma management], [support provided by the stoma clinic], and [compromising with defecation dysfunctions] (Table 5). With respect to [coping with defecation dysfunctions], one participant stated *“I took magnesium oxide, as necessary, after starting the anticancer drugs. I took too much magnesium oxide, which caused severe diarrhea, and had to go to the washroom about 12 times per day. Sometimes I had severe diarrhea after taking the anticancer drugs. The home helper tells me to ‘keep hydrated’ during every visit*”; this reflected <bowel control for constipation and diarrhea>. Another participant stated “*I had more than 10 events of leakage*. *When I don’t wear a diaper,* *I often mess myself,”* which indicated <coping with anal incontinence>.

[Perceived difficulty in stoma management] encompassed <concerns about the sound of gas and the bulge caused by the stoma appliance> and <concerns about fecal odor and leakage>. One participant stated “*What bothers me most is fecal leakage from the stoma bag*. *It would be a bit easier if I had no problems with fecal leakage. That bothers me*”; this indicated that fecal odor and leakage are psychological burdens for the participant.

[Support provided by the stoma clinic] consisted of <care for stoma complications> and <pelvic floor muscle training after stoma closure>. Regarding the former, one participant who visited the stoma clinic for stoma management stated “*I have pelvic floor muscle training in the stoma clinic. I mistook cancer for diarrhea and suffered for 10 months. The tumor enlarged and almost occluded the anal canal. Compared to that suffering, the current stoma appliance is fine*.”

[Compromising with defecation dysfunctions] comprised of <conflict in the adaptation process> due to <changes in body image and reduced self-esteem>. One participant with <family and social support> stated *“This is the reality. I think the treatment saved my life. The best thing was the fact that cancer helped me realize that I am happy,”* indicating that such participants were compromising with defecation dysfunctions through <gratitude for the treatment>.

### 3.5. Survey of Exercise Habits

In the non-stoma group, nine subcategories of exercise habits were identified and combined into two broad categories: [maintenance of exercise habits] and [perceived difficulty of exercise due to reduced physical fitness after surgery] (Table 4). Regarding [maintenance of exercise habits], one participant stated *“I used to play table tennis for up to 3 h a day, about* *3 times a week. I play lawn tennis. I am still a leader of a lawn tennis club. I am a member of a tennis club in the town,”* reflecting maintenance of exercise habits appropriate for a patient’s postoperative physical condition due to <preoperative exercise habits>, <absence of pain>, and <a positive attitude toward exercise>.

Regarding [perceived difficulty of exercise due to reduced physical fitness after surgery], one participant had <awareness of reduced physical function due to aging or surgery> and claimed “*I have systemic problems. It is even difficult to crouch. I cannot even stand up and sit down quickly as I did when I was young*,” reflecting <perceived difficulty of postoperative exercise>. Additionally, the participant stated “*Anticancer drugs caused severe side effects. I had numbness and substantial pain in the limbs*. *COVID-19 made everything difficult*,” reflecting a fear of infection and <exercise limitation due to the side effects of chemotherapy> (neuropathy and increased susceptibility). Another participant stated “*I do not know what to use and how to use the tools for home exercise. It would make life easier if they taught me about the predictors of physical function in patients undergoing rehabilitation for recovery. I do not even know the predictors of recovery of physical function. What I vaguely remember is how mobile I was,”* reflecting a <lack of information on postoperative predictors of exercise and available social resources>.

In the stoma group, five categories of exercise habits were identified; these were combined into two broad categories: [maintenance of exercise habits] and [perceived difficulty of exercise after colostomy] (Table 5). [Maintenance of exercise habits] consisted of <preoperative exercise habits> and a <positive attitude toward exercise>. One participant stated *“I regularly walk because it is good for my health. Although it may sound strange, I walk about 40 min a day as a hobby. I walk about 5,000 steps a day,”* indicating that <preoperative exercise habits> were maintained even after surgery. [Perceived difficulty of exercise after colostomy] consisted of <awareness of the handicap>, <sensation of pressure and discomfort due to the stoma>, <exercise dysfunction due to pain or neuropathy>, and <exercise limitation due to reduced physical fitness or chemotherapy> (Table 5). With respect to becoming a person with disabilities, one participant said *“After all, I am handicapped because the stoma is compressed when crouching or picking stuff up,”* reflecting <perceived difficulty of exercise>. Additionally, another participant stated “*I hate that I can only walk slowly because of exercise dysfunction caused by pain or neuropathy due to comorbidity and chemotherapy. I hate that I became a bit weak. I like sports but not extreme ones. I had so many things that I wanted to accomplish, but now it is impossible for me,”* reflecting <change in exercise habits due to reduced physical fitness>.

## 4. Discussion

In this qualitative lifestyle study of defecatory dysfunction and postoperative exercise habits among survivors of rectal cancer, we could compare and contrast the characteristics of patients in both the non-stoma and stoma groups. Four categories common to the two groups were identified: [diet control], [coping with defecation dysfunctions], [compromising with defecation dysfunctions], and [maintenance of exercise habits]. In contrast, two categories were identified for survivors without pre- and postoperative exercise habits: [difficulty coping with defecation dysfunctions] and [perceived difficulty of exercise after colostomy]. 

In a systematic review of 25 cases, Neuberger et al. [9] concluded that stoma insertion negatively affects physical function and quality of life in older people. In our study, the EORTC QLQ-30 survey results indicated that the non-stoma group had a higher QoL than the stoma group.

### 4.1. Comparison of Defecation Dysfunction and Exercise Habits by Stoma Status

The participants in this study used [diet control] to [cope with defecation dysfunctions]. Previous studies found that dietary fiber ameliorates diarrhea, constipation, and fecal incontinence [4], suggesting that certain eating habits play a role in preventing ileus and maintaining postoperative bowel control.

Dietary management is the most common approach for ameliorating defecatory dysfunction. However, this approach requires clinical guidelines because patients’ decisions so far have been based on their own experiences and assumptions; as such, the relationship between food types and defecation patterns remains uncertain [10]. These issues are important to address because improving physical activity and eating habits from the perspective of patient self-management reportedly prolongs survival [11]; therefore, an approach that includes both diet and physical activity rather than either one alone is crucial.

Additionally, [compromising with defecation dysfunctions] led to [maintenance of exercise habits]. In contrast, those in the non-stoma group who did not exercise either before or after surgery had a seven-fold higher frequency of bowel movements and [difficulty coping with defecation dysfunctions], suggesting that defecation dysfunctions due to low anterior resection syndrome [12] negatively impacts exercise habits. Pelvic floor muscle training has been reported to improve fecal incontinence [5]; in contrast, only a few studies have investigated the positive effects of aerobic and resistance exercise.

Our current study revealed that, in the non-stoma group, <lack of information on postoperative predictors of exercise and available social resources> and <lack of information on defecation dysfunctions> were issues of concern, indicating a need for more specific guidance regarding exercise. Moreover, <awareness of reduced physical function due to aging or surgery> was identified as a problem in the non-stoma group. In contrast, <change in exercise habits due to reduced physical fitness> was a concern in the stoma group. These findings suggests that exercise intervention is necessary to prevent a decline in physical function. Furthermore, Neuberger et al. [9] reported that appropriate exercise patterns and regular walking alleviated defecatory dysfunction. Therefore, additional studies of defecatory dysfunction and exercise habits in survivors with and without stomas are warrented.

This study demonstrated that the [perceived difficulty of exercise after colostomy] caused by <exercise dysfunction due to pain or neuropathy> was common for those who did not exercise before or after surgery and those who discontinued exercise postoperatively. Asnong et al. [13] investigated physical activity levels in 120 survivors during the 12 months following low anterior resection. They found that the presence of a stoma was a predictor of low physical activity levels. Responses such as <awareness of the handicap> and <a sensation of pressure and discomfort due to the stoma> also showed the adverse effects of colostomy on postoperative exercise habits. However, four participants in the stoma group began to exercise within 5 years of surgery despite not having exercised preoperatively suggests a change in behavior owing to <a positive attitude toward exercise> and <changes in exercise habits due to reduced physical fitness>. Furthermore, the exercise duration per week (post-surgery) exceeded the 150 min recommended in the exercise guidelines for cancer survivors [14], thereby exemplifying health awareness and the long-term self-management of bowel dysfunction using exercise [15]. 

Collectively, our findings illustrated the importance of a safer and more feasible combined intervention program incorporating eating, bowel movement, and exercise habits by identifying predictors of exercise based on physical activities [8] and guidelines.

### 4.2. QoL Trends in the Presence or Absence of a Stoma

The lower QoL in the stoma group may stem from emotional distress likely owing to changes in excretion habits because of the presence of a colostomy bag and defecation dysfunction. Additionally, patients in the stoma group experienced higher levels of fatigue and pain than did those in the non-stoma group on the symptom scales. These results conform to [perceived difficulty of exercise after colostomy] consisting of <exercise dysfunction due to pain or neuropathy> as identified during our interviews. A systematic review and meta-analysis of 19 trials on the effect of exercise among individuals with colorectal cancer by Singh et al. [16] found that exercise led to improvements in QoL, vigor, aerobic fitness, upper-body strength, cheerfulness, sleep, and body weight. Exercise improves the effectiveness of treatment for cancer-related fatigue during and after surgery for non-advanced colorectal cancer [17]. The results of this study suggest that 30% of the participants who did not exercise, especially elderly patients with stomas who had reduced physical fitness after surgery, require interventions for symptom relief and exercise. 

### 4.3. Strengths and Limitations

We first evaluated defecation dysfunction and exercise habits in survivors of colorectal cancer with and without stomas. The study strength lies in its novelty, and our findings may provide fundamental clinical evidence to promote nutrition education and exercise habits in survivors with postoperative defecation dysfunction. Interventions for exercise habit formation may also improve the QoL [16]. However, the study had some limitations. First, our findings may not be generalizable because only 17 survivors participated. Second, the presence or absence of stomas was a possible confounding factor due to different excretion pathways and their effects on QoL (although this was evaluated using the EORTC QLQ-C30, which allows for direct comparisons). Pachier et al. [7] did not find that the QoL of patients with stomas was lower than that of patients without, which is inconsistent with our findings. The reasons for this discrepancy may be: (i) only patients with rectal cancer were included in our study, and (ii) the mean postoperative follow-up duration for the stoma group was 1 year. Additional studies with larger sample sizes are therefore warranted. The findings allow us to design a new questionnaire for a large-scale quantitative study to develop a combined program to improve eating habits and ameliorate defecation dysfunction.

## 5. Conclusions

Our survey of lifestyle habits among rectal cancer survivors with and without stomas found that the participants engaged in [diet control] to [cope with defecation dysfunctions] and [maintained exercise habits] by [compromising with defecation dysfunctions]. Those without [maintenance of exercise habits] had [difficulty coping with defecation dysfunctions] and experienced [perceived difficulty in stoma management].

Our findings suggest the need for providing interventions to improve eating and exercise habits to maintain physical function and alleviate defecatory dysfunction in survivors of rectal cancer, including those with and without stomas.

## Figures and Tables

**Table 1:** Participant characteristics.

Item		Non-Stoma (*n* = 6)	Stoma (*n* = 11)
Sex	Male	5 (83.3%)	9 (81.8%)
	Female	1 (16.7%)	2 (18.2%)
Age (years)	Male	75.8 ± 8.6	65.1 ± 14.6
	Female	67.0 ± 0	68.5 ± 6.4
Smoking		0 (0%)	2 (18.2%)
BMI	(kg/m^2^)	22.0 ± 1.7	22.5 ± 4.0
HbA1c	(%)	6.2 ± 0.5	6.3 ± 1.2
Diagnosis	Rectal cancer	6 (35.3%)	11 (64.7%)
TNM-UICC stage	IIa	3 (50.0%)	3 (27.3%)
	IIb	0 (0%)	1 (9.1%)
	IIIa	1 (16.7%)	2 (18.2%)
	IIIb	2 (33.3%)	1 (9.1%)
	IV	0 (0%)	4 (36.4%)
Operative procedure	APE/Hartmann	0 (0%)	6 (54.5%)
	LAR	6 (35.3%)	5 (45.5%)
Stoma	Colostomy	-	6 (54.5%)
	Ileostomy	-	5 (45.5%)
Postoperative period (years)		2.5 ± 1.1	1.0 ± 1.6
Medical history	Diabetes mellitus	0 (0%)	4 (36.4%)
	Hypertension	1 (16.7%)	2 (18.2%)
	Stroke	1 (16.7%)	2 (18.2%)
	Angina pectoris	1 (16.7%)	1 (9.1%)
	Gallstones	1 (16.7%)	1 (9.1%)
	Liver abscess	0 (0%)	1 (9.1%)
	Ileus	1 (16.7%)	3 (27.3%)
	Osteoarthrosis	0 (0%)	1 (9.1%)
Stoma complications	Parastomal hernia	-	1 (9.1%)
	Abdominal incisional hernia	1 (16.7%)	0 (0%)
	Peristomal dermatitis	-	3 (27.3%)
Chemotherapy		3 (50%)	4 (36.4%)
Radiotherapy		0 (0%)	0 (0%)

Data are presented as *n* (%) or mean ± standard deviation. Administration of chemotherapy and radiotherapy 3 months before registration was examined. TNM-UICC, Tumor-node-metastasis staging per the Union for International Cancer Control; BMI, body mass index; HbA1c, hemoglobin A1c; APE, abdominoperineal excision; LAR, low anterior resection.

**Table 2:** Overview of pre- and postoperative lifestyles.

Lifestyle Items			Non-Stoma (*n* = 6)	Stoma (*n* = 11)
Postoperative weight gain (kg)			1.3 ± 1.2	2.3 ± 2.0
Preoperative exercise habits	Yes		5 (83.3%)	5 (45.5%)
Postoperative exercise habits	Yes		5 (83.3%)	7 (63.6%)
Preoperative duration of exercise/week (min)			101.1 ± 73.3	75.5 ± 145.0
Postoperative duration of exercise/week (min)			286.7 ± 201.4	185.5 ± 169.1
Preoperative duration of exercise per session	≥60 min		3 (50%)	3 (27.3%)
Postoperative duration of exercise per session	≥60 min		4 (66.7%)	2 (18.2%)
Preoperative exercise frequency/week	No		1 (16.7%)	7 (63.6%)
	<3		2 (33.3%)	1 (9.1%)
	≥3		3 (50%)	3 (27.3%)
Postoperative exercise frequency/week	No		1 (16.7%)	4 (36.4%)
	<3		1 (16.7%)	0 (0%)
	≥3		4 (66.6%)	7 (63.6%)
Postoperative exercise content	Walking	(3.5)	3 (50.0%)	6 (54.5%)
(METs)	Aerobiking	(3.5)	0 (0%)	2 (18.2%)
	Table tennis	(4.0)	1 (16.7%)	0 (0%)
	Tennis	(4.5)	1 (16.7%)	0 (0%)
	Ground golf	(3.5)	1 (16.7%)	0 (0%)
	Yoga	(2.5)	0 (0%)	1 (9.1%)
	Exercise	(4.5)	1 (16.7%)	1 (9.1%)
	Pelvic floor muscle exercise	(2.3)	0 (0%)	3 (27.3%)
Pre- and postoperative activities of daily living	Housework	(3.3)	2 (33.3%)	2 (18.2%)
(METs)	Home gardening	(3.5)	0 (0%)	1 (9.1%)
	Work and commuting	(3.5)	2 (33.3%)	1 (9.1%)
	Dog-walking	(3.0)	1 (16.7%)	0 (0%)
Meal frequency/day	Two times		0 (0%)	1 (9.1%)
	Three times		6 (100%)	10 (90.9%)
Nausea	Yes		0 (0%)	0 (0%)
	No		6 (100%)	11 (100%)
Stool frequency/day	Before surgery		1.3 ± 0.8	2.8 ± 2.2
	After surgery		2.5 ± 1.5	-
Frequency of discarding stoma excretion/day			-	3.5 ± 2.0
Laxative	Yes		2 (33.3%)	2 (18.2%)
Antidiarrheal agent	Yes		2 (33.3%)	0 (0%)

Data are presented as *n* (%) or mean ± standard deviation. Metabolic equivalents (METs) for postoperative exercise content and regular pre- and postoperative physical activity were calculated according to the 2011 Compendium of Physical Activities [8].

**Table 3:** QoL evaluated using the EORTC QLQ-30 (version 3).

Item		Non-Stoma	Stoma
Functional scales	Physical functioning	90	53
	Role functioning	93	67
	Emotional functioning	93	70
	Cognitive functioning	77	73
	Social functioning	63	63
Symptom scales	Fatigue	0	40
	Nausea and vomiting	3	0
	Pain	0	5
	Dyspnea	0	27
	Insomnia	0	40
	Appetite loss	0	20
	Constipation	33	23
	Diarrhea	10	13
	Financial difficulties	0	30
Summary score		90	70
Global health status/QoL		83	40

The summary score and global health status/QoL score were calculated according to the EORTC QLQ-C30 Scoring Manual (2001) [6]. EORTC-QLQ-C30, European Organisation for Research and Treatment of Cancer’ QoL questionnaire-30; QoL, quality of life.

**Table 4:** Categories of lifestyle impacts in the non-stoma group.

	Category	Subcategory
Eating habits	Diet control	Carbohydrate and calorie restrictions to normalize blood glucose levels
		Water and dietary intake considering excretion
		Utilization of information on postoperative diet
	Changes in appetite	Anorexia as a side effect of chemotherapy
		Recovery of body weight
Bowel habits	Coping with defecation dysfunctions	Understanding of defecation rhythm using a stool diary
		Carrying disposable diapers for fecal incontinence
	Difficulty coping with defecation dysfunctions	Dissatisfaction with bowel control
		Lack of information on defecation dysfunctions
	Compromising with defecation dysfunctions	Regret their delayed visit to the hospital and express gratitude for obtaining treatment
		Adaptation to defecation dysfunctions
Exercise habits	Maintenance of exercise habits	Preoperative exercise habits
		Absence of pain
		Positive attitude toward exercise
	Perceived difficulty in postoperative exercise	Awareness of reduced physical function due to aging or surgery
		Exercise limitation due to the side effects of chemotherapy (neuropathy and increased susceptibility)
		Lack of information on postoperative predictors of exercise and available social resources

**Table 5:** Categories of lifestyle impacts in the stoma group.

	Category	Subcategory
Eating habits	Diet control	Avoiding foods high in insoluble fiber
		Dietary control after colostomy
		Utilization of information on postoperative diet
	Taste disorders as a side effect of chemotherapy	Taste disorders caused by chemotherapy
		Weight loss due to reduced dietary intake
Bowel habits	Coping with defecation dysfunctions	Bowel control of constipation and diarrhea
		Coping with anal incontinence
	Perceived difficulty in stoma management	Concerns about the sound of gas and the bulge caused by the stoma appliance
		Concerns about fecal odor and leakage
	Support provided by the stoma clinic	Care for stoma complications
		Pelvic floor muscle training after stoma closure
	Compromising with defecation dysfunctions	Changes in body image and reduced self-esteem
		Conflict in the adaptation process
		Family and social support
		Gratitude for receiving treatment
Exercise habits	Maintenance of exercise habits	Preoperative exercise habits
		Positive attitude toward exercise
	Perceived difficulty in exercise after colostomy	Awareness of becoming handicapped
		A sensation of pressure and discomfort caused by the stoma
		Exercise dysfunction due to pain or neuropathy
		Change in exercise habits due to reduced physical fitness

## Data Availability

The study data are available on request from the corresponding author.
